# Dopamine Transporter Knockout Rats Show Impaired Wellbeing in a Multimodal Severity Assessment Approach

**DOI:** 10.3389/fnbeh.2022.924603

**Published:** 2022-07-11

**Authors:** Anne Stephanie Mallien, Laura Becker, Natascha Pfeiffer, Federica Terraneo, Melissa Hahn, Anthonieke Middelman, Rupert Palme, Kerstin Camile Creutzberg, Veronica Begni, Marco Andrea Riva, Damiana Leo, Heidrun Potschka, Fabio Fumagalli, Judith R. Homberg, Peter Gass

**Affiliations:** ^1^Department of Psychiatry and Psychotherapy, RG Animal Models in Psychiatry, Central Institute of Mental Health, Medical Faculty Mannheim, Heidelberg University, Mannheim, Germany; ^2^Department of Pharmacological and Biomolecular Sciences, Università degli Studi di Milano, Milan, Italy; ^3^Department of Cognitive Neuroscience, Centre for Neuroscience, Donders Institute for Brain, Cognition, and Behaviour, Radboud University Medical Center, Nijmegen, Netherlands; ^4^Department of Biomedical Sciences, University of Veterinary Medicine, Vienna, Austria; ^5^Department of Neurosciences, University of Mons, Mons, Belgium; ^6^Institute of Pharmacology, Toxicology, and Pharmacy, Ludwig-Maximilians-University (LMU), Munich, Germany

**Keywords:** rat model, dopamine, psychiatry, animal welfare, severity assessment, behavior, endophenotypes

## Abstract

In preclinical psychiatry research, animals are central to modeling and understanding biological mechanisms of behavior and psychiatric disorders. We here present the first multimodal severity assessment of a genetically modified rat strain used in psychiatric research, lacking the dopamine transporter (DAT) gene and showing endophenotypes of several dopamine-associated disorders. Absence of the DAT leads to high extracellular dopamine (DA) levels and has been associated with locomotor hyperactivity, compulsive behaviors and stereotypies in the past. The German Animal Welfare Law, which is based on the EU Directive (2010/63/EU), requires a prospective severity assessment for every animal experiment, depending on the extent of the expected degree of pain, suffering, distress or lasting harm that the animals will experience. This should consider all procedures but also the impact of the genotype on the phenotype. Therefore, we examined multiple parameters indicating animal welfare, like burrowing behavior, social interaction, saccharin preference, baseline stress hormone levels and nesting behavior. Additionally, a footprint analysis was performed and home cage activity was analyzed for a more detailed characterization of locomotion. DAT KO rats demonstrated reduced burrowing, social interaction and saccharin preference. We also found pronounced stereotypies and alterations in the gait analysis in DAT KO rats. Moreover, we confirmed the hyperactivity and the impaired sensorimotor gating mechanisms to assure that our rats are exhibiting the correct phenotype. In conclusion, we provide evidence that DAT KO rats show alterations in natural behavior patterns and deduce that the marked stereotypies are a sign for coping difficulties, both indicating a negative influence of the genotype on wellbeing. We suggest to assess further rat models in an objectified severity assessment as previously done in mice to create a relative severity assessment based on scientific evidence. Until then, we propose the classification of homozygous DAT KO rats as “moderate” in accordance with the criteria of the EU directive 2010/63.

## Introduction

Since the implementation of the EU Directive 2010/63 every member state of the European Union is obliged to classify the severity of every planned animal experiment into “non-recovery,” “mild,” “moderate,” or “severe” before conduction. The category depends on the degree of pain, suffering, distress or lasting harm that is expected to be experienced by an animal during the experiment. This classification is important for the harm-benefit analysis which is used as basis for ethical justification of the experiment. Appendix VIII of the Directive provides examples for the severity classification of selected procedures and serves as a guideline for determining the extent and duration of pain, suffering, distress or lasting harm emerging from the entire experiment, taking procedures and the genotype into consideration. Up to today, the EU Directive does not provide detailed descriptions of “mild,” “moderate,” or “severe” phenotype manifestations.

Replacement is one major goal of the 3R principle (Russell and Burch, 1959), but continuing *in vivo* research is still necessary in the field of neuroscience because of lacking alternatives to animal experimentation, in particular for questions concerning behavior (Homberg et al., 2021). Hence, it is necessary to focus even more on the other 2Rs–reduction and refinement–to continuously optimize animal research. Refinement are any means to reduce suffering or increase wellbeing in the animals. Importantly, wellbeing does not only comprise the absence of negative experiences but also the presence of positive experiences. Therefore, an evaluation of the *status quo* regarding the affective state of laboratory animals is mandatory. For that purpose, in a national research project with partners across Germany, a framework for severity assessment has been established (Bleich and Tolba, 2017). Multiple parameters have been proposed to assess the internal state of the rodents (Jirkof et al., 2019; Keubler et al., 2020). We here present a first multimodal severity assessment of a genetically altered rat strain used in psychiatry.

A dopamine transporter (DAT) KO rat line was recently developed using zinc-finger nucleases (ZFN) technology, leading to deletion of the *SLC6A3* gene, which codes for the DAT (Leo et al., 2018b). The DAT controls dopamine (DA) levels in the synaptic cleft by transporting released DA back into the dopaminergic neuron and is an essential molecule in modulation of DA levels (Klein et al., 2019). As a consequence, the DAT KO rats have increased basal extracellular DA levels with an enormously extended half-life and subsequently reduced intracellular DA storage levels measured by fast-scan cyclic voltammetry (Leo et al., 2018b). Dopaminergic signaling plays a key role in controlling locomotion systems, motivation, cognition, reward and pleasure (Gainetdinov, 2008; Salamone and Correa, 2012; Klein et al., 2019). In previous studies DAT KO rats showed locomotor hyperactivity, cognitive dysfunctions and deficits in sensorimotor gating, as well as traits of compulsive behavior (Cinque et al., 2018; Leo et al., 2018b)–endophenotypes found in psychiatric disorders associated with altered dopaminergic signaling like attention deficit hyperactivity disorder (ADHD), obsessive-compulsive disorder (OCD), bipolar disorder and schizophrenia (Leo et al., 2018a). Therefore, they present an important experimental tool to gain fundamental insights in the biological mechanisms of these disorders.

In the present study, we aimed to assess the wellbeing of DAT KO rats. To assemble the necessary information about the affective state for an evidence-based severity assessment, we examined a repertoire of natural rodent behaviors, comprising burrowing, preference for sweet solutions and nest-building activity. Because of previous reports on hyperactivity in DAT KO rats, we also further characterized the changes in locomotion by applying non-invasive home-cage monitoring and footprint analyses. We also studied physiological parameters, such as body weight, food and water intake, baseline corticosterone concentrations and applied a modified Irwin test as neurobehavioral score. Corticosterone metabolites were measured in the feces of the animals to avoid stress induced by taking blood samples. This non-invasive method is validated in rats and helps to provide insight into stress hormone response (Lepschy et al., 2007). Results from our study are pivotal to classify the phenotype in a severity category and to develop future refinement measures.

## Materials and Methods

### Animals

Dopamine transporter knock-out (DAT KO) rats were generated as described elsewhere (Leo et al., 2018b). In the present study we used 16 male homozygous DAT KO, 20 heterozygous DAT (DAT HET) and 20 wildtype (WT) rats bred on Wistar-Han background. All rats were bred in the animal facility of the Radboud University (Nijmegen, Netherlands) and transported to the Central Institute of Mental Health (CIMH, Mannheim, Germany) at the age of 8–9 weeks. At CIMH animals were pair-housed with cage mates of the same genotype in conventional Type IV cages (59,8 cm × 38 cm, 20 cm, length × width × height; Tecniplast, Hohenpeißenberg, Germany) in a room with a 12:12 h light-dark cycle (lights on: 7 a.m.) and controlled room temperature (22 ± 1°C) and humidity (45 ± 5%). The cages were equipped with bedding (Abedd Espen MIDI, ABEDD, Vienna, Austria), cardboard tunnels (Zoonlab, Castrop-Rauxel, Germany), gnawing sticks (Zoonlab), paper and sizzle nest (Zoonlab) for enrichment and were changed weekly. Food (LASQCdiet Rod16, Altromin Spezialfutter GmbH & Co. KG, Lage, Germany) and tap water were given *ad libitum* and the intakes were measured weekly. All experiments were performed according to the regulations for animal experimentation in the European Union (European Communities Council Directive 2010/63/EU) and in the German Animal Welfare Act and were approved by the German animal welfare authorities (Regierungspräsidium Karlsruhe, 35-9185-81-G-143-19). Reporting follows the checklist of the ARRIVE guidelines 2.0 (Percie du Sert et al., 2020). Behavioral testing started after the animals had been habituated for 12 days. During habituation rats were handled daily and daily body weight measures were taken as well.

### Experimental Plan

**FIGURE 1:**
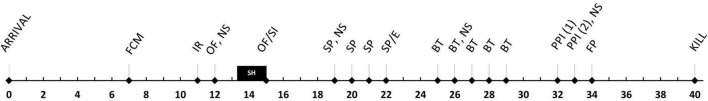
Timeline. Numbers indicate the day of the experiment. FCM, fecal corticosterone metabolites; IR, irwin test; OF, open field; NS, nest score; SH, single housing; SI, social interaction; SP, saccharin preference; E, ethogram; BT, burrowing test; PPI, prepulse inhibition; P, footprint analysis.

All behavioral testing took place during the light phase between 9 a.m. and 3 p.m., unless otherwise stated. Two separate cohorts performed each test, both were handled by the same experimenters. The timeline is shown in Figure 1. The order of the tests was chosen so that low-stress experiments were performed before the potentially more stressful ones in order to limit confounding effects due to sequential testing.

Wellbeing of the rats was evaluated by the burrowing test, a social interaction test, nest scoring and a two-bottle choice paradigm for anhedonia. These parameters have been suggested to be suited for severity assessment (Deacon, 2006; Keubler et al., 2020; Schwabe et al., 2020; van Dijk et al., 2020). To further characterize the previously reported hyperactivity of the DAT KO rats we also analyzed spontaneous behavior in the home cage during the animals’ active phase and studied differences in gait by footprint analyses. We also assessed basic physiological parameters, like body weight, water and food intake, as well as fecal corticosterone metabolites. Moreover, we applied a modified Irwin test as a neurobehavioral score. Lastly, to confirm the validity of the DAT KO rat model, we aimed to confirm their typical behavioral alterations (hyperactivity, sensory gating alterations) in our laboratory. For that purpose we conducted an open field test (OF), the acoustic startle response (ASR) and prepulse inhibition (PPI) test.

The experimenters were blinded to the genotypes. However, DAT KO animals were often directly distinguishable from the others by their smaller body size.

#### Welfare Parameters

##### Burrowing Test

We assessed the burrowing behavior of the rats based on a protocol slightly modified from Deacon (2006). The rats were trained for 5 days. On the first day each rat was habituated for 30 min to a Type IV cage with tissue covering the floor. On the consecutive days, the rats received an empty burrowing tube for 30 min, before a plastic burrowing tube (32 cm long × 10 cm in diameter, open end 6 cm elevated from the floor) filled with gravel (2,000 g) was placed in the cage for 60 min and the rats were allowed to burrow. Then, the amount of burrowed gravel was measured. On day 5 we additionally measured the latency to burrowing for 600 s. Rats, that did not start burrowing in these 600 s, we used 600 s for descriptive and comparative statistical analysis. Between trials the gravel was washed with 0.1% acetic acid. The rest of the equipment was cleaned with 70% ethanol.

##### Saccharin Preference

A preference for a sweetened solution with saccharin is normal for rats that can choose between water and the sweet solution. A missing preference is indicative for an anhedonic state of the animals (Willner et al., 1987). The protocol was tested over four consecutive days according to Klein et al. (2015). Briefly, water consumption for comparison was tested on day 1 and 3. On day 2 and 4 one water bottle was replaced by a similar bottle filled with 0.1% saccharin (Sigma-Aldrich Chemie GmbH, Taufkirchen, Deutschland) solution, alternating the position on both days. 24 h consumption values were determined by weighing the bottles daily at the same time. The saccharin preference was calculated using the following formula: $\frac{c\,o\,n\,s\,u\,m\,e\,d\,s\,a\,c\,c\,h\,a\,r\,i\,n\,s\,o\,l\,u\,t\,i\,o\,n\,\left(m\,l\right)*100}{t\,o\,t\,a\,l\,f\,l\,u\,i\,d\,c\,o\,n\,s\,u\,m\,p\,t\,i\,o\,n\,\left(m\,l\right)}$. To avoid the position of the bottle as a confounding factor, all cages were equipped with two water bottles during the entire experiment.

##### Social Interaction

Analyzing social behavior of rats has been shown to be an informative parameter to determine severity in rats (van Dijk et al., 2020). We modified this protocol, separating the rats from their cage mates for only 48 h in conventional Type III cages (42,5 cm × 27,6 cm × 15,3 cm; length × width × height; Tecniplast, Hohenpeißenberg, Germany) with an elevated lid according to the specifications in the EU Directive 2010/63. During the test, the animals were reunited with their former cage mate in the open field apparatus, after habituation to the experimental room for 10 min. The two rats that were tested simultaneously always had the same genotype. Each rat’s behavior was scored from videos in active and passive interactions for 5 min, using a modified protocol according to Schneider et al. (2016). Active interactions were scored when the rat either showed contact behavior (social grooming or crawling over/under the partner’s body) or social exploration, further divided in investigating the anogenital or a non-anogenital region of the partner or approaching/following of the partner. Passive interactions were scored when the tested rat displayed inert behavior toward the social partner initiating social contact (Schneider et al., 2016). We scored the frequency of interactions as well as the time (duration) in the first 5 min of re-introducing the rats. Afterward, the rats returned to their previous housing condition.

##### Nest Score

Starting from week 2 we assessed nest building performance according to the nest scoring scheme published by Schwabe et al. (2020). The score distinguishes between the scores 0 to 4, with score 0 representing almost untouched nest material and score 4 describing the highest nest complexity. The nest scoring scheme considers interaction with nest material, its distribution on the cage floor, visibility of a nesting area, the shape of the edges as well as the height, respectively, the indentation of the nest (Schwabe et al., 2020). Twenty-four hours after providing fresh nesting material during the weekly cage change we took photos, showing the nests from above, which were then analyzed retrospectively. Therefore, the raters were first trained by scoring a first batch of nests and then discussing the obtained scores in order to standardize scoring. After the training four raters scored the nest scores of the first cohort and three raters scored the ones of the second cohort.

#### Further Characterization of Locomotion

##### Ethogram

To compare unevoked behaviors of the animals in an undisturbed state, we observed each animal for 10 min in their active phase (7:30 p.m.–10 p.m.) under red light conditions. We scored the behavioral patterns every 30 s and grouped them into the following categories: self-care, inactive, active, stereotypies and social. Self-care was scored when the rat exhibited either eating, drinking, self grooming or nest building. Inactive behavior included sleeping only, whereas active behaviors consisted of walking, jumping, digging, rearing, sniffing or climbing. We classified circling, “weaving” and bar mouthing as stereotypies. Weaving was defined as a stereotypic movement of the front body from left to right and *vice versa* and was evident in the DAT KO rats even before conducting the ethogram. “Social” is self-explaining and included all shown social interactions.

##### Footprint Analysis

Therefore, we used the footprint analysis protocol as reported by Carter et al. (2001). Briefly, the front and back paws were marked with different colors of non-toxic paint and the rats were encouraged to run through a corridor that was covered with paper. The corridor was narrow enough to ensure the rats could run one-way through it only. We then measured front and hind base, fore- and hindlimb stride as well as the overlap between forepaw and hindpaw placement on the paper as described (Carter et al., 2001).

#### Physiology

##### Water and Food Intake

The water intake of the animals was measured by weighing the water bottles weekly at the same time of the day. The food in the cage lid was weighed and refilled once a week.

##### Fecal Corticosterone Metabolite Analysis

We assessed baseline FCM levels for each rat to investigate if DAT deletion leads to altered stress hormone balance under baseline conditions. For that purpose, the animals were intermittently single housed in conventional Type III cages (Tecniplast, Hohenpeißenberg, Germany) for 2 h (10 p.m.–12 p.m.). Feces were collected from the cages on the next morning and frozen at −20°C. The samples were further processed as described in Sensini et al. (2020). Briefly, aliquots (50 mg) were taken from each dried and homogenated fecal sample and mixed with 1 ml of 80% methanol. The methanol extract was shaken for 30 min and centrifuged for 10 min before taking the supernatant for analysis using the 5α-pregnane-3β,11β,21-triol-20-one enzyme immunoassay established by Lepschy et al. (2007). All samples were handled equally concerning temperature.

##### Modified Irwin Test

We used a modified version of the Irwin test (for details see Supplementary Material) to detect general neurobehavioral deviations between DAT KO and DAT HET and wildtype control rats (Irwin, 1968; Moller et al., 2018).

#### Activity and Prepulse Inhibition Phenotyping

##### Open Field

Animals were tested in the open field test for 60 min to assess baseline locomotor behavior. Prior to testing rats were habituated for at least 10 min to the experimental room. The open field apparatus consisted of four adjacent gray boxes (50 cm in length × 50 cm in width × 50 cm in height) dimly lit with 25 lux. The equipment was wiped with 70% ethanol between trials. Parameters analyzed were total distance moved (TDM), center time, movement and velocity, and were tracked automatically in 5 min time bins (Ethovision XT, Noldus Information Technology, Wageningen, Netherlands) (Chourbaji et al., 2005). Because of technical problems during recording only data of one cohort could be analyzed.

##### Acoustic Startle Response and Prepulse Inhibition

Acoustic startle response and PPI were evaluated in startle chambers (SR-LAB, San Diego Instruments, San Diego, CA, United States) as previously described (Schneider and Spanagel, 2008). The program started with 5 min background noise (65 dB). The ASR was evoked by a 115 dB sound signal. For determination of PPI, prepulses at four different intensities (72, 76, 80, and 84 dB) were played 100 ms prior to the startle signal. PPI is the attenuation of the startle response after a preceding less intense prepulse signal. The program conducted the trials in a pseudorandom order and each trial type (background noise, startle stimulus, prepulse stimuli with different intensities) was presented for 10 s. PPI is reported in percentage (attenuated mean startle amplitude with prior prepulse/mean amplitude without prepulse). After each testing session the apparatus was cleaned with 70% ethanol. For logistic reasons the testing took place over two consecutive days, each animal was tested only once. Twelve animals (WT *n* = 3, HET *n* = 5, KO *n* = 4) had to be excluded from analysis because the testing tube was not in the correct position.

### Statistics

Statistical analysis was performed using IBM SPSS statistics 27. One-Way ANOVA and repeated measures ANOVA were performed unless stated otherwise and if the data matched the required qualifications (normal distribution, homoscedacity). *Post-hoc* analysis was adjusted with Bonferroni correction to correct for multiple testing. If applicable, “cohort” was used as a covariate to test for possible cohort effects. Welch ANOVA was performed in case of normal distributed data with heteroscedacity. *Post-hoc* analysis was then calculated using Games Howell correction. Non-parametric testing was performed using Kruskal–Wallis Test. Kendall’s coefficient of concordance W was used to calculate inter-rater reliability in the nest score. We calculated the Spearman correlation coefficient to investigate for possible relationships between burrowing, saccharin preference, active interactions in the social preference task, TDM in the open field and FCM concentration. The significance level was set to α < 0.05. The statistical unit was the individual animal, except for the saccharin preference test, water consumption and the nest score, where the cage was used as statistical unit. Unless stated otherwise, all elevated data points were included in the statistical analysis. *A priori* G*power analyses were conducted to calculate sample sizes on basis of estimated effect sizes for one-way ANOVA. Assumption was made on previously reported effect sizes or experience in our laboratory. The power was set to 80% and the type I error to 5%. We calculated a sample size of *n* = 16 per genotype as a minimum.

## Results

### Dopamine Transporter Knock-Out Rats Do Not Burrow, Show Less Preference for Saccharin and Indicate Reduced Social Interaction

**FIGURE 2:**
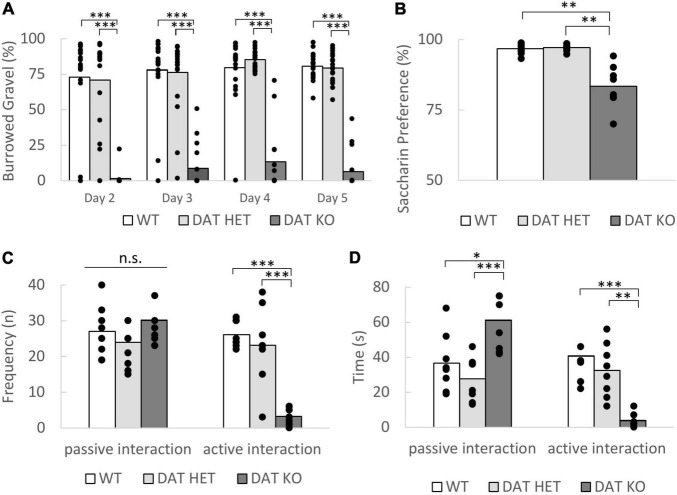
**(A–D)** Wellbeing parameters. **(A)** Burrowing test. The burrowed gravel in percentage over the training week is expressed as the mean. The DAT KO rats burrowed significantly lower amounts of gravel on all days. Sample Size: DAT KO (*n* = 16), DAT HET (*n* = 20), WT (*n* = 20). **(B)** Saccharin preference test. The bars show the mean preference of the saccharin solution over water over both testing days. The preference for the saccharin solution is significantly lower for the DAT KO rats. Sample Size: DAT KO (*n* = 8), DAT HET (*n* = 10) and WT rats (*n* = 10). **(C,D)** Social interaction. **(C)** Frequency of passive and active interactions shown during the first 5 min of the social interaction test. The DAT KO rats displayed less active interactions compared to the DAT HET and WT rats. No differences were observed in the frequency of passive interactions. **(D)** Duration of the shown active and passive interactions shown during the first 5 min of the social interaction test. The DAT KO rats spent significantly more time interacting passively and less time with active interactions compared to the two other genotypes. **(C,D)** Data are expressed as the mean + SEM. Sample size for all genotypes *n* = 8. **(A–D)** The dots in the bar graphs indicate all included data points in the analysis. *p < 0.05, ^**^p < 0.01, and ^***^p < 0.001.

In the burrowing test DAT KO rats showed a significantly reduced burrowing behavior when compared to the heterozygous and wildtype rats [Figure 2A, repeated measures ANOVA, genotype effect, *F*_(2,52)_ = 139.692, *p* < 0.001, *post-hoc* (Bonferroni) KO vs. HET *p* < 0.001, KO vs. WT *p* < 0.001]. They also revealed a prolonged latency to burrow [one-way ANOVA, genotype: *F*_(2,52)_ = 5.273, *p* = 0.008; *post-hoc* (Bonferroni) KO vs. WT *p* = 0.043, KO vs. HET *p* = 0.009, data not shown] on day 5 of the training. Although not measured quantitatively, we observed stereotypical behaviors such as bar mouthing, excessive running and gnawing on the tubes instead of burrowing in the DAT KO. Surprisingly, the DAT HET and WT rats already exhibited a very high burrowing performance on the first day, which increased only slightly over the remaining testing days.

Interestingly, the DAT KO showed a significant lower preference for the saccharin solution [Figure 2B, Welch ANOVA *F*_(2, 12.86)_ = 12.51, *p* = 0.001, *post-hoc* (Games Howell): KO vs. WT *p* = 0.003, KO vs. HET *p* = 0.003]. This confirms the findings by Cinque et al. (2018).

During the social interaction test, we observed that both the frequency and time spent with active interactions is much lower in DAT KO rats than in DAT HET and WT rats [Figures 2C,D, one-way ANOVA, frequency *F*_(2,21)_ = 26.788, *p* < 0.001, *post-hoc* (Bonferroni) KO vs. HET *p* < 0.001, KO vs. WT *p* < 0.001; time *F*_(2,21)_ = 11.885, *p* = 0.0004, *post-hoc* (Bonferroni) KO vs. HET *p* = 0.005, KO vs. WT *p* < 0.001]. There was no significant difference in the frequency of passive interactions [genotype: *F*_(2,21)_ = 1.067, *p* = 0.362], but a difference in the total duration of passive interaction [one-way ANOVA *F*_(2,21)_ = 9.980, *p* = 0.0009, *post-hoc* (Bonferroni) KO vs. HET *p* < 0.001, KO vs. WT *p* = 0.014]. Whereas data from WT and DAT HET were in a comparable range, DAT KO rats spent much more time on passive interactive behavior.

We did not observe significant group differences in the nest score in any week of the experiment (data not shown). The nest scores were quite low (median 1–2) in all groups at all time points. This low nest building performance might result from the enriched cage environment. We observed that the rats often used the cardboard tunnels as nests and therefore might not have had a strong motivation to build additional nests. The inter-rater reliability calculated using Kendall’s coefficient of concordance was *W* = 0.731 in one cohort (4 raters) and *W* = 0.693 in the other cohort (3 raters).

No correlations were detected between the parameters burrowing, saccharin preference, active interactions in the social preference task, TDM in the open field and FCM concentration (data not shown).

### Dopamine Transporter Knock-Out Rats Show Altered Gait Patterns and Reduced Activity

**FIGURE 3:**
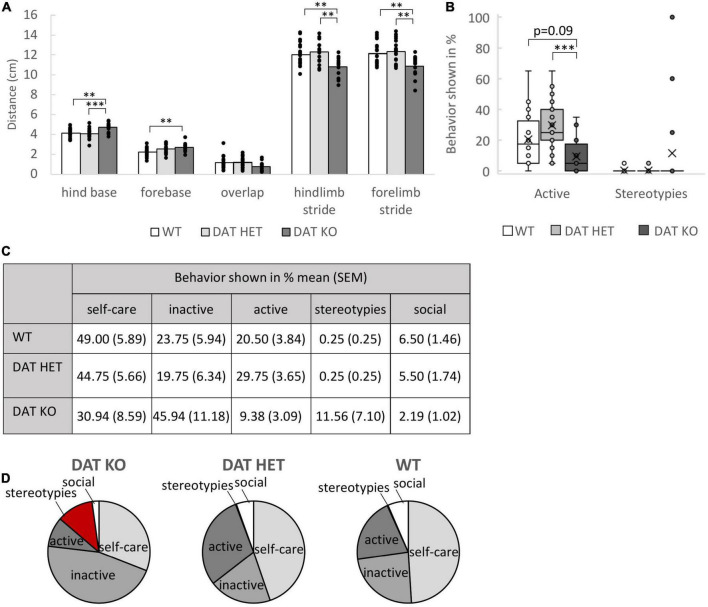
**(A–D)** Footprint Analysis and Ethogram. **(A)** Footprint analysis. The bars present the mean length in cm for the different parameters in the footprint analysis and the dots represent all data points. The DAT KO rats had a shorter forelimb as well as hindlimb stride. The hind base (vs. DAT HET and WT) and the fore base (vs. WT) were significantly broader in the DAT KO rats. Sample size: DAT KO (*n* = 16), DAT HET (*n* = 20), WT (*n* = 20). **(B)** Boxplots illustrating the distribution of the categories “active” and “stereotypies” during the 10 min home-cage observation for the three genotypes. **(C)** Table presenting the distribution of the categorized behaviors. **(D)** Pie charts showing the distribution of the categorized behaviors for the different genotypes. **(B,D)** Ethogram. The DAT KO rats spent less time with active behaviors, such as walking, jumping, digging, rearing, sniffing or climbing, compared to DAT HET and WT rats. They also showed a higher percentage for stereotypical behaviors (circling, “rocking” and bar mouthing), although there was no genotype effect. Pronounced stereotypic behavior was shown by individual DAT KO rats (boxplot). Sample size: DAT KO (*n* = 16), DAT HET (*n* = 20), and WT rats (*n* = 20). **(A-D)**: ^**^p < 0.01, ^***^p < 0.001.

Footprint analyses demonstrated an altered gait pattern of DAT KO rats as compared to DAT HET and WT controls (Figure 3A). One-way ANOVA revealed a reduced forelimb stride [*F*_(2,51)_ = 8.855, *p* = 0.001, *post-hoc* (Bonferroni) KO vs. WT *p* = 0.005, KO vs. HET *p* = 0.001] as well as a reduced hindlimb stride [*F*_(2,51)_ = 9.954, *p* < 0.001, *post-hoc* (Bonferroni) KO vs. WT *p* = 0.005, KO vs. HET *p* = 0.001] and an increased hind base [*F*_(2,51)_ = 9.954, *p* < 0.001, *post-hoc* (Bonferroni) KO vs. WT *p* = 0.002, KO vs. HET *p* < 0.001], reminding of a frog’s gait. Fore base was only different between DAT KO and WT rats [*F*_(2,51)_ = 6.593, *p* = 0.003, *post-hoc* (Bonferroni) KO vs. WT *p* = 0.003, KO vs. HET *p* = 0.718].

Observing the rats in their active phase for 10 min revealed that only selected rats from the DAT KO group exhibited repeated stereotypical behavioral patterns (Figures 3B–D). This finding was based on the behavior of 3 conspicuous rats (Figure 3B). Thus, data analysis did not confirm significant group differences for stereotypic behavior. Non-parametric Kruskal–Wallis Testing revealed a genotype effect only in the active behaviors of the rats [Kruskal Wallis, H(2) = 14.347, *p* = 0.001, *post-hoc* (Bonferroni) KO vs. WT *p* = 0.086, KO vs. HET *p* < 0.001].

### Dopamine Transporter Knock-Out Rats Weigh Less, Drink More but Show no Difference in Fecal Corticosterone Metabolites

**FIGURE 4:**
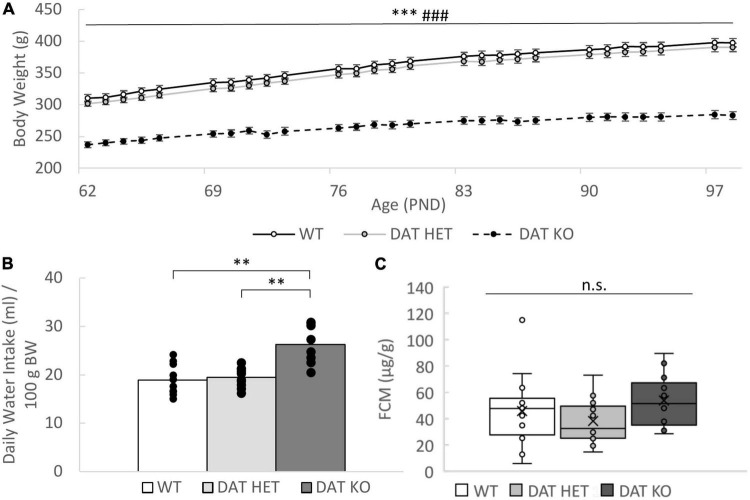
**(A–C)** Physiological Parameters. **(A)** Body Weight. Development of the body weight during the experiment. The DAT KO rats had a significant lower body weight throughout the experiment. Data shown are expressed as the mean. Sample size: DAT KO (*n* = 16), DAT HET (*n* = 20), WT (*n* = 20). PND, postnatal day. **(B)** Water Intake. Bars illustrate the mean daily water intake per 100 g body weight measured weekly (Saccharin preference test week was excluded). The dots indicate all elevated data points included. The DAT KO rats drink significantly lower amounts of water compared to the other genotypes. Sample Size: DAT KO (*n* = 7), DAT HET (*n* = 9), WT (*n* = 8). **(C)** Fecal corticosterone metabolite (FCM) concentration. Boxplots showing the FCM concentrations for the three genotypes. No genotype effect was observed. Sample size: DAT KO (*n* = 13), DAT HET (*n* = 13), WT (*n* = 15). **(A–C)** ^**^p < 0.01, ^***^p < 0.001 between HET and KO, ### *p* < 0.001 between WT and KO.

Our study confirms the previous findings that the body weight of DAT KO rats is significantly lower compared to the other genotypes during the entire experiment [genotype: *F*_(2,52)_ = 77.083, *p* < 0.001, *post-hoc* (Bonferroni) KO vs. WT *p* < 0.001, KO vs. HET *p* < 0.001, Figure 4A]. This finding is in line with previous reports by Cinque et al. (2018) and Leo et al. (2018b). We also confirmed a flatter growth curve in DAT KO rats [time*genotype interaction: *F*_(56,52)_ = 31.397, *p* < 0.001].

Throughout the experiment DAT KO rats drank more water in relation to their body weight than the other genotypes [one-way ANOVA, genotype: *F*_(2,10)_ = 24.1, *p* < 0.001, *post-hoc* (Bonferroni) KO vs. HET *p* < 0.001, KO vs. WT *p* < 0.001, Figure 4B]. The week of the saccharin preference test was excluded from the analysis of water consumption due to lacking comparability. Cinque et al. (2018) found no difference in the water consumption regarding the total fluid intake. No difference in food consumption was found [one-way ANOVA, genotype: *F*_(2,24)_ = 0.085, *p* = 0.919].

No significant group differences were detected in the Irwin Test [Kruskal–Wallis Test; H(2) = 4.309, *p* = 0.116]. Nevertheless, we observed a higher variance in DAT KO rats than for DAT HET and WT rats resulting from higher scores in individual DAT KO rats (see Supplementary Material). Altered parameters in DAT KO rats were increased locomotor activity, touch-induced escaping, defecation while handling, increased body tone while handling, stereotypies, vocalizations and a reduced startle behavior.

Baseline FCM concentrations showed no significant differences between treatment groups [*F*_(2,37)_ = 1.445, *p* = 0.249, Figure 4C], indicating that DAT KO rats do not have an elevated level of stress hormones in general. Since not all animals defecated during the 2 h sampling period, the sample size was slightly reduced (KO *n* = 13, HET *n* = 13, WT *n* = 15).

### Dopamine Transporter Knock-Out Show Typical Hyperactivity and Sensory-Gating Alterations

The behavior of the DAT KO rats in the open field confirmed the previously described hyperactivity and stereotypical circling (see Supplementary Material). Additionally, analysis in DAT KO rats displayed reduced PPI (see Supplementary Material).

## Discussion

The present study was designed to determine the intrinsic burden of the recently developed DAT KO rat strain. For that purpose, we investigated several natural rodent behaviors commonly used for severity assessment (Jirkof et al., 2019). We found a reduction of burrowing behavior as well as reduced social interactions and a mild anhedonia in the saccharin preference test. Further characterization of the previously described locomotor alterations (i.e., hyperactivity) (Cinque et al., 2018; Leo et al., 2018b; Adinolfi et al., 2019; Reinwald et al., 2022) revealed an abnormal gait pattern in the footprint analysis as well as reduced activity during home-cage monitoring. Additionally, by examining physiological parameters we were able to demonstrate increased water intake, equal corticosterone levels, and lower body weight in DAT-KO rats compared to matched controls. The neurobehavioral scoring scheme using a modified Irwin test showed more variability in DAT KO rats. However, total scores did not differ between groups. Lastly, we also confirmed previously reported behavioral alterations, i.e., hyperactivity in the open field and altered sensorimotor gating in DAT KO rats (Cinque et al., 2018; Leo et al., 2018b; Adinolfi et al., 2019; Reinwald et al., 2022). This confirmation was important to ensure that we are measuring the burden of the anticipated phenotype including all reported alterations.

### Compromised Wellbeing in Dopamine Transporter Knock-Out Rats

One definition of animal welfare described it as not only “the absence of negative experience” but also the “presence of positive experiences” (Boissy et al., 2007). During the course of this study we found two major criteria affecting wellbeing in the DAT KO rats: (1) DAT KO rats presented a lack of natural rodent behaviors as well as mild anhedonia and (2) they displayed marked stereotypies in multiple experiments.

(1) DA plays a crucial role in reward and motivation *via* the mesocorticolimbic system (Klein et al., 2019). A previous study on DAT KO rats suggested an impaired reward system. Similar to our study, Cinque et al. (2018) showed that DAT KO rats had a mild anhedonic phenotype in the saccharin preference test and additionally seemed less interested in eating mascarpone cheese. Burrowing behavior was suggested to comprise self-rewarding properties, which might play a role in the decreased interest in burrowing (Deacon, 2006; Klein et al., 2019). It is a behavior that usually should be natural to rodents and thought to be indicative for negative affective states if absent (Jirkof et al., 2019). Previous studies could link reduced burrowing performance for example to impaired welfare in rat models for epilepsia (Koska et al., 2019; Seiffert et al., 2019; van Dijk et al., 2020). DAT KO rats almost did not burrow at all, but showed stereotypical behaviors instead. Furthermore, burrowing was proposed to be a sensitive parameter to detect neurobehavioral toxicity (Deacon, 2009). The low burrowing performance in the DAT KO rats could therefore also result from excess dopamine levels in the synaptic cleft possibly causing neuronal cell death (Cadet et al., 2010).

The social behavior of DAT KO in our study was impaired. One cause for the reduced social behavior might have been the overall hyperactive and distracted phenotype shown by the DAT KO when facing new contexts such as the open field box in which social interaction was assessed. Although not significant, the home-cage observation revealed also lower counts of social behaviors in DAT KO rats. Given that rats are highly social animals (Schweinfurth, 2020), this again indicated at least impaired natural behaviors and therefore reduced wellbeing.

In a study by Vengeliene et al. (2017), DAT deficient rats also revealed less social behavior in a social interaction test. Surprisingly, Adinolfi et al. (2019) showed aberrant social behavior of the DAT HET rats only in a social preference task, characterized by rapid onset of inactive behavior compared to WT animals.

(2) Our observations confirm stereotypy findings (Cinque et al., 2018; Adinolfi et al., 2019). In captive animals stereotypies have been often associated with low welfare and have been suggested to reflect poor housing conditions (Wurbel, 2001; Mason et al., 2007). Perseverative behaviors are suggested by Mason and Latham (2004) to not always be associated with reduced subjective wellbeing by drawing a link to patients diagnosed with autism spectrum disorder. Self-reports have revealed that some patients sometimes perceive repetitive behaviors as pleasuring or calming (Joyce et al., 2017). Patients diagnosed with OCD instead sense repetitive behaviors as bothering and unwanted (Postorino et al., 2017). Anyway, perseveration tendencies in changing environmental conditions seem to adversely affect coping abilities. In DAT KO rats this became apparent, whenever they were faced with novel stimuli (e.g., open field box, new cages for burrowing) and immediately responded with stereotypical behaviors. Importantly, the stereotypies even persisted over natural rodent behavior like burrowing and the noise deriving from it in the neighboring cages. Even after habituation to the experimental setup and the task for several days they showed no interest in the provided burrowing material. The rats could be distressed by changed factors, e.g., by a short-time social instability as well as a new environment. Resulting from that, we state that the impaired coping abilities in DAT KO rats lead to reduced natural behaviors, again indicating reduced wellbeing. Future research is needed to find an innovative enrichment specifically for rats showing this type of behavior to increase the phenotype’s ability to cope with novel environments during experiments.

In our study neither of the genotypes showed reduced nest building behavior. A decrease of nest-building and therefore lower nest complexity scores indicate a reduced wellbeing of the animals (Jirkof, 2014; Schwabe et al., 2020). Whereas nest-building has been shown to be a reliable parameter for assessing the wellbeing of mice (Jirkof, 2014; Hager et al., 2015), there is less evidence for rats, although this parameter was successfully implemented in one rat model of epilepsia (Moller et al., 2018). In other severity assessment studies nest-building in rats was not found to be a very reliable parameter and the models assessed were sometimes considered stressful on basis of other parameters (Koska et al., 2019; Riedesel et al., 2021). In contrast to Schwabe et al. (2020) we equipped the cages with tissue and a cardboard tube in addition to the sizzle nesting material, which might have been too much material in the cage also rendering scoring difficult. In future approaches we suggest to use less enrichment during the time of nest scoring to improve the sensitivity of the test.

### Activity and Locomotion

Marked hyperactivity was confirmed demonstrating the robustness and reproducibility of earlier findings. A striking observation in the open field was that DAT KO rats displayed repetitive circular movements, similar to multiple other cohorts rats tested in our lab (Reinwald et al., 2022). In a structural and functional MRI study, Reinwald et al. (2022) have linked the hyperactive and repetitive behavior of the DAT KO rats to volume alterations in the striatum and cerebellum, directly resulting from the genetic modifications (i.e., high extracellular dopamine levels) of the animals. We performed a gait analysis:whereas the shorter stride lengths of the DAT KO rats could easily be explained by the smaller size, the wider hind and fore base could be caused by (1) excessive running [similar to orthopedic changes in mice that run excessively in the wheel (Richter et al., 2014)], (2) a compensatory mechanism to maintain balance as seen in ataxia-associated gait patterns (Kyriakou et al., 2016) or (3) an anatomic misalignment resulting from the homozygous genotype [e.g., dwarfism in the hypothalamic growth hormone releasing hormone (Bosse et al., 1997)] or combination of these factors. Whether the wider base is a burden to the animal is not clear. Further examinations should clarify if early onset of joint degeneration and consecutive painful arthritis are occurring, particularly for breeding colonies and studies in older animals. However, this can be circumvented by mating DAT HET.

Interestingly, while observing the rats in their home cages in the dark phase, approximately 0.5–3 h after switching off the light, the only genotype-associated alterations was hypoactivity in DAT KO rats, whereas parameters as stereotypic behaviors, self-care and inactive behaviors did not significantly differ from controls. Twenty-four hours spontaneous locomotor activity recordings in the home cage performed by Leo et al. (2018b) demonstrated, that DAT KO rats did not show increased locomotion compared to WT rats in the last hours of the light phase and the first hours in the dark phase. This indicates that DAT KO rats did not exhibit hyperactivity throughout the day and exhibited normal resting states. Interestingly, we were unable to detect a reduction in locomotion in the afternoon while testing the rats in the open field (conduction: 9 a.m. to 4 p.m.). Unlike the home-cage based approach, our rats were faced with an environmental change (i.e., transfer in open field boxes) which was likely to reduce resting behavior in that time period in a genotype-independent manner.

### Physiological Data

Dopamine transporter knock-out rats weighed less and showed a flattened growth curve during the entire experiment. As already stated by Cinque et al. (2018) this could be either related to hyperlocomotion or because of decreased levels of growth hormone in DAT KO mice, or a combination of both (Bosse et al., 1997). The DAT KO rats consumed significantly more water in relation to their body weight, whereas food consumption did not differ between genotypes. We assumed that the DAT KO rats consume more water because of their hyperactivity and subsequent higher metabolic rate. The lack of differences in FCM concentration argues against relevant alterations in baseline hypothalamus-pituitary-adrenal (HPA) axis activity in DAT KO rats. Measurement of FCMs is a non-invasive technique to approximate stress hormone levels without interfering with the endocrine response (Palme, 2019). An interesting future approach would be the measurement of stress hormones after HPA axis activation induced by stress in DAT KO rats. The modified Irwin test was unremarkable except for higher variance in the DAT KO rats, indicating no fundamental differences in neurobehavioral scores.

### Dopamine Transporter Knock-Out as a Model for Psychiatric Disorders

Multiple studies have examined the DAT KO rats and have discussed endophenotypic features for ADHD, obsessive compulsive disorder (OCD), schizophrenia and bipolar disorder. For example Cinque et al. (2018) observed rigid choice patterns and compulsive behaviors in an intolerance-to-delay task, suggesting that the rat model recapitulates phenotypic features of ADHD and OCD. Adinolfi et al. (2019) also discovered compulsive behaviors in a marble burrowing task, increased arousal in a social-preference, reduced anxiety in the dark-light box and a longer latency to freeze in a fear conditioning paradigm displayed by DAT KO rats. Leo et al. (2018b) reported an impaired working memory and hyperactivity in an open field test that was reversible by amphetamines. However, in a schedule-induced polydipsia DAT KO rats lacked compulsive behaviors in contrast to controls. In previous and our present study a reduced PPI was detected in DAT KO rats (Leo et al., 2018b; Reinwald et al., 2022). This condition is also described for patients diagnosed with schizophrenia, obsessive compulsive disorder and ADHD (Braff et al., 1978; Braff et al., 2001; Ahmari et al., 2012), diseases that are linked to altered dopaminergic signaling (Klein et al., 2019). Although the DAT KO rats do not model one specific psychiatric disorder, they comprise multiple endophenotypes of disorders linked to altered dopaminergic signaling (see above). Many patients suffering from ADHD, OCD or schizophrenia are reporting a lower quality of life (Solanki et al., 2008; Quintero et al., 2019; Remmerswaal et al., 2020). From an anthropomorphic view one could imagine that animals, modeling psychopathologies, could also resemble patients in regard to their burden. However, it is impossible to know whether the rats really have similar experiences or perceptions as patients. Also, patients often not only experience the symptoms of their disease, but also social stigmatization, which further exacerbates their life quality. We do not expect such complications in our experimental setup. However, we can get estimates of the animals’ wellbeing by their physiology and alterations in behavior and derive an objective assessment of severity from their natural behavioral traits.

## Conclusion

Taken together, our results revealed that knockout of the DAT in rats led to restrictions of natural rodent behaviors, including aberrant social interactions, almost complete lack of burrowing behavior and reduction of anhedonia-associated behavior. Together with the displayed stereotypies, which were especially pronounced in new environments, our findings led us conclude that DAT KO rats show a reduced wellbeing due to coping difficulties when facing new situations. Therefore, we would like to emphasize the importance to further identify and validate parameters to assess the wellbeing of animals, especially in the psychiatric research context and to examine the *status quo* of animal models used in this research field. We also suggest an investigation of enrichment on coping abilities as a future refinement approach.

For an ideal classification of the severity grade of the DAT KO rats, we would suggest a systematic approach as already proposed for mice (Talbot et al., 2020). By applying the same wellbeing parameters in many rat models, sensitive and comparative evidence-based severity assessment will become feasible. This would finally guarantee an objectified mechanism to assess the burden of our animals. Until this is possible, we would suggest a severity classification of the DAT KO rats as “moderate” because their shown phenotype presented permanent mild behavioral alterations as well as short-term moderate distress when exposed to novel environments.

## Data Availability Statement

The original contributions presented in this study are included in the article/Supplementary Material, further inquiries can be directed to the corresponding author/s.

## Ethics Statement

The animal study was reviewed and approved by the Regierungspräsidium Karlsruhe.

## Author Contributions

ASM, HP, FF, JH, DL, RP, and PG: conceptualization. ASM, LB, and NP: formal analysis. RP, DL, HP, JH, and PG: funding acquisition. ASM, LB, NP, FT, MH, KC, and VB: investigation. ASM, AM, and PG: project administration. RP, MR, HP, FF, JH, and PG: resources. ASM, KC, HP, and PG: supervision. ASM, LB, NP, FT, MH, AM, RP, KC, VB, MR, DL, HP, FF, JH, and PG: validation and writing – review and editing. LB and ASM: Visualization. LB, ASM, and PG: roles/writing – original draft. All authors contributed to the article and approved the submitted version.

## Conflict of Interest

The authors declare that the research was conducted in the absence of any commercial or financial relationships that could be construed as a potential conflict of interest.

## Publisher’s Note

All claims expressed in this article are solely those of the authors and do not necessarily represent those of their affiliated organizations, or those of the publisher, the editors and the reviewers. Any product that may be evaluated in this article, or claim that may be made by its manufacturer, is not guaranteed or endorsed by the publisher.
