# Supplementary material for: Dopamine Transporter Knockout Rats Show Impaired Wellbeing in a Multimodal Severity Assessment Approach
Source: Front Behav Neurosci. 2022 Jul 11;16:924603. doi: 10.3389/fnbeh.2022.924603 (PMC9309546; doi:10.3389/fnbeh.2022.924603)
Supplement: Supplementary file 1 [file Data_Sheet_1.docx]

Supplementary Material

## Methods

**Supplementary Table 1:** Parameters of the modified Irwin Test as reported in (Moller et al., 2018).

| Number | Parameter | Meaning |
| --- | --- | --- |
| 1 | Body position | -2 flat, lying on the side or on the belly, no muscle tone  -1 partially preserved muscle tone, occasional postural corrections  0 normal posture  +1 rigid or cramped posture (e.g., Opisthotonus)  +2 animals show no resting position, therefore cannot be evaluated |
| 2 | Ptosis | 0 eyelids open normally  +1 eyelid ½ open  +2 eyelids closed |
| 3 | Locomotor activity | -2 no activity  -1 diminished movement, occasional cleaning  0 normal activity  +1 increased, powerful, angular movements  +2 restless motion type |
| 4 | Touch-response | -2 failure, even stronger stimuli remain without reaction  -1 after multiple or enhanced stimulus slow, weakened reaction (ducking, escaping)  0 normal response to light touch  +1 dodge or duck on light touch  +2 escape over greater distance, attack behavior, vocalization to light touch |
| 5 | Grasp-irritability | 0 none, +1 slight, +2 marked |
| 6 | Curiosity (showing object) | -2 no reaction  -1 decreased curiosity  0 normal, interest in presented object  +1 jerky tracking, no adaptation even with repeated withdrawal  +2 attack Behavior |
| 7 | Provoked freezing | 0 none  +1 slight  +2 marked, abrupt freeze |
| 8 | Vocalization | 0 none  +1 spontaneous low pitched beeps  +2 more spontaneously occurring stronger beeps |
| 9 | Urination  (while handling) | 0 none  +1 present |
| 10 | Defecation  (while handling) | 0 none  +1 present |
| 11 | Lid reflex | 0 normal  +2 impacted |
| 12 | Corneal reflex | 0 normal  +2 impacted |
| 13 | Startle | -2 no reaction  -1 delayed or less reaction  0 normal  +1 escape  +2 strong reaction, closing of the eyes, attaching the ears, pressing on the floor or temporary freezing |
| 14 | Pelvic elevation | -1 flattened  0 normal  +1 slightly increased |
| 15 | Tail elevation | -1 regrinding the tail while walking  0 normal tail position  +1 inclined tail (maximum 90 ° angle) during resting and activity period, partial tail hitting  +2 tails over the body (Straub phenomenon) |
| 16 | Limb rotation | 0 not present  +1 slight  +2 marked |
| 17 | Body tone (while handling) | -2 completely flaccid  -1 low tonus  0 normal muscle tone  +1 increased tonus  +2 total body stiffness |
| 18 | Abdominal tone | -1 reduced muscle tone  0 normal  +1 increased muscle tension |
| 19 | Righting reflex (if animal lies continuous) | -2 not present  -1 reduced  0 no impairment |
| 20 | Ataxia | 0 no ataxia  +1 coordinating problems during running  +2 loss of motion coordination |
| 21 | Exophthalmos | 0 no Exophthalmia  +1 mild exophthalmia  +2 pronounced exophthalmia |
| 22 | Hypersalivation | 0 none  +1 present |
| 23 | Lacrimation | 0 none  +2 present |
| 24 | Feces | -1 no or hardly any feces in the cage  0 feces and quantity normal  +1 feces significantly increased  +2 almost liquid feces, diarrhea |
| 25 | Piloerection | 0 none  +2 present |
| 26 | Skin perfusion | -2 tail and paws bluish-discolored: cyanosis  -1 almost white pinna, paws or tails with barely visible vessels  0 normal skin perfusion  +1 paws dark pink, tail strong light pink  +2 paws, pinna or tail red |
| 27 | Respiratory rate | -2 snapping, acute respiratory distress  -1 decreased, irregular, strained breathing  0 normal breathing  +1 increased breathing rate  +2 as 3, but also during resting |
| 28 | Tremors | 0 none  +2 available |
| 29 | Twitches | 0 none  +2 available |
| 30 | Convulsions | 0 none  +2 available (describe the type) |
| 31 | Area of implant | -2 high-grade necrotic changes in the implant area  -1 slight necrotic changes in the implant area  0 normal implant area  +1 minor inflammation in the implant area  +2 high-grade inflammation in the implant area |
| 32 | Stereotypies | 0 none  +2 available |
| 33 | Body temperature | -1 temperature by 2 °C or more against the control  0 temperature normal  +1 temperature is increased by 2 °C or more against the control |

## Results

**DAT KO show typical hyperactivity and sensory-gating alterations**

The behavior of the DAT KO rats in the open field largely parallels results of already published data (Cinque et al., 2018; Leo et al., 2018; Adinolfi et al., 2019; Reinwald, in press). Similarly, we observed marked hyperactivity, as indicated e.g. by the total distance moved (TDM) (***Supplemental Fig. 1*A**) and movement (***Supplemental Fig. 1*D**). Repeated measures ANOVA revealed a genotype effect and a time*genotype effect (genotype: F(2,21)=91.688, p<0.001, post hoc (Bonferroni) KO vs. WT p<0.001, KO vs. HET p<0.001; time*genotype interaction: F(22,231)=12.471, p<0.001) for TDM. In contrast to the other genotypes, the homozygous DAT KO rats increased their moved distance over the testing time and kept it at a constant high level over the last 40 min (***Supplemental Fig. 1*A**). As the DAT KO animals seem prone to stereotypical behaviors, we created heatmaps summarizing the places of stay over the total time (***Supplemental Fig. 1*D**). Consistent to a previous study undertaken in our lab (Reinwald, in press), we observed the homozygous DAT KO animals moving in repetitive circles.

DAT KO rats displayed a higher center time (***Supplemental Fig. 1*E**, genotype: F(2,21)=8.670, p=0.002, post hoc (Bonferroni) KO vs. WT p<0.006, KO vs. HET p<0.004). The behavior of the DAT KO animals is further accompanied by a higher velocity (***Supplemental Fig. 1*C**, genotype: F(2,21)=89.745, p<0.001, post hoc (Bonferroni) KO vs. HET p<0.001, KO vs. WT p<0.001) and a higher percentage in movement (***Supplemental Fig. 1*D**, genotype: F(2,21)=266.490, p<0.001, post hoc (Bonferroni) KO vs. WT p<0.001, KO vs. HET p<0.001) compared to the other genotypes.

In the Acoustic Startle Response (ASR) no genotype effect was noticeable (***Supplemental Fig. 1*G**, one-way ANOVA; genotype: F(2,40)=2.329, p=0.11). Repeated measures ANOVA confirmed the expected intensity effect of the prepulse and a genotype effect (intensity: F(3,120)=9.861, p<0.001, genotype: F(2,40)=10.137, p<0.001, post hoc (Bonferroni): KO vs. HET p=0.001, KO vs. WT p<0.001), shown in ***Supplemental Fig. 1*H**. No cohort effects could be detected.

## Figures


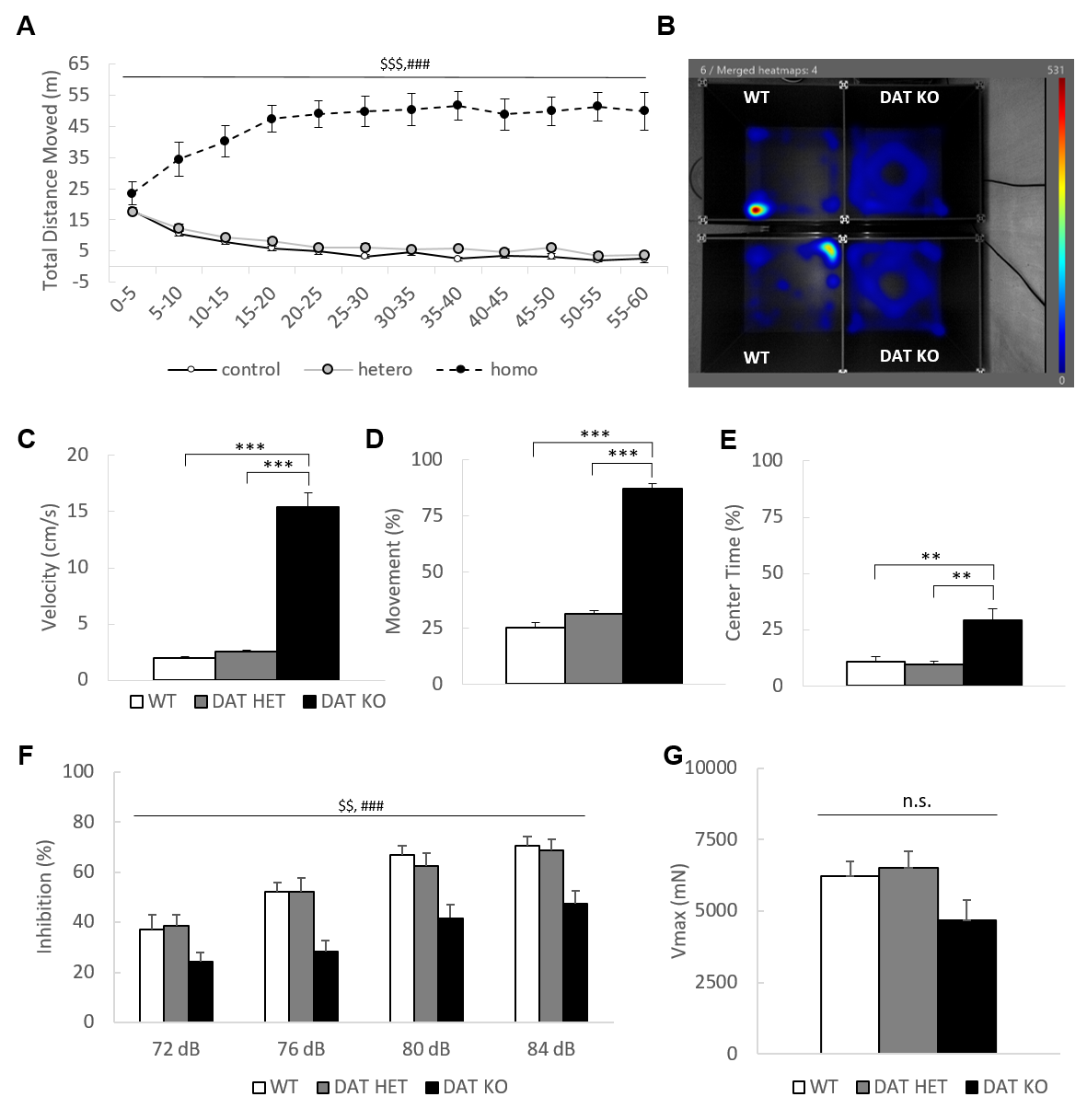


Supplemental Fig. 1*(*A-G): Activity and PPI Phenotyping. (A-E) Open field analysis. (A) Total distance moved in cm measured in 5-min time bins over the time course of 60 min. DAT KO rats covered a greater distance during all time periods compared to DAT HET and WT rats. (B) Heat map of the movement of two representative DAT KO and WT rats, respectively, summed up over 60 min. The DAT KO rats displayed repetitive circling over the entire time, whereas the WT rats also spent longer times at a location. (C) Mean velocity in cm/s shown during the total 60 min of the open field test. The DAT KO rats displayed a higher velocity than the DAT HET and WT rats. (D) Percentage of movement of the animals during the total 60 min. The relative movement percentage is significantly higher for the DAT KO rats. (E) Percentage of time spent in the center of the open field test during the total 60 min. The DAT KO rats spent significantly more time in the center of the OF than the other genotypes. (A-E) Sample size for all genotypes was n=8. (F-G) Prepulse inhibition and acoustic startle respons. (F) Bars show the intensity of the inhibition of the startle response after presenting a prepulse (72 dB, 76 dB, 80 dB and 84 dB). The DAT KO rats had a significantly weaker prepulse inhibition over all PPI intensities compared to DAT HET and WT rats. (G) The bars indicate the mean + SEM of the V_max_ in the ASR. No genotype effect was apparent in the acoustic startle response. (F-G) Sample size: DAT KO (n=12), DAT HET (n=15), WT (n=17). A-G: * p<0.05, ** p<0.01, *** p<0.001, $$ p<0.01/$$$ p<0.001 between KO and HET, ### p<0.001 between KO and WT.


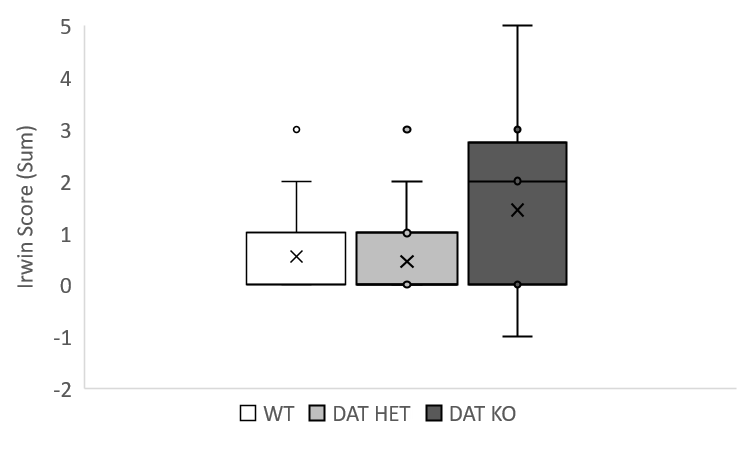


Supplemental Fig. 2: Irwin Scores. Boxplots indicate the sums of the modified Irwin test. No significant group differences were apparent. DAT KO (n=16), DAT HET (n=20), WT (n=20).

**References:**

Adinolfi A, Zelli S, Leo D, Carbone C, Mus L, Illiano P, Alleva E, Gainetdinov R R, Adriani W (2019). Behavioral characterization of DAT-KO rats and evidence of asocial-like phenotypes in DAT-HET rats: The potential involvement of norepinephrine system. Behavioural Brain Research, 359: 516-527

Cinque S, Zoratto F, Poleggi A, Leo D, Cerniglia L, Cimino S, Tambelli R, Alleva E, Gainetdinov R R, Laviola G, Adriani W (2018). Behavioral Phenotyping of Dopamine Transporter Knockout Rats: Compulsive Traits, Motor Stereotypies, and Anhedonia. Front Psychiatry, 9: 43

Leo D, Sukhanov I, Zoratto F, Illiano P, Caffino L, Sanna F, Messa G, Emanuele M, Esposito A, Dorofeikova M, Budygin E A, Mus L, Efimova E V, Niello M, Espinoza S, Sotnikova T D, Hoener M C, Laviola G, Fumagalli F, Adriani W, Gainetdinov R R (2018). Pronounced Hyperactivity, Cognitive Dysfunctions, and BDNF Dysregulation in Dopamine Transporter Knock-out Rats. The Journal of Neuroscience, 38(8): 1959-1972

Moller C, Wolf F, Van Dijk R M, Di Liberto V, Russmann V, Keck M, Palme R, Hellweg R, Gass P, Otzdorff C, Potschka H (2018). Toward evidence-based severity assessment in rat models with repeated seizures: I. Electrical kindling. Epilepsia, 59(4): 765-777

Reinwald J R G, N.; Mallien, A.S.; Sartorius, A.; Becker, R.; Sack, M.; Falfan-Melgoza, C.; Clemm Von Hohenberg, C.; Leo, D.; Pfeiffer, N.; Middelman, A.; Meyer-Lindenberg, A.; Homberg, J.R.; Weber-Fahr, W.; Gass, P. (in press). Dopamine Transporter Silencing in the Rat: Systems-level Alterations in Striato-Cerebellar and Prefrontal-Midbrain Circuits. Molecular Psychiatry,
